# Non-Specific Binding and Cross-Reaction of ELISA: A Case Study of Porcine Hemoglobin Detection

**DOI:** 10.3390/foods10081708

**Published:** 2021-07-23

**Authors:** Xingyi Jiang, Meng Wu, Jonathan Albo, Qinchun Rao

**Affiliations:** 1 Department of Nutrition and Integrative Physiology, Florida State University, Tallahassee, FL 32306, USA; xj15@my.fsu.edu; 2 Institute of Biology, Hebei Academy of Sciences, Shijiazhuang 050081, China; biobang@sina.com; 3 Meinig School of Biomedical Engineering, Cornell University, Ithaca, NY 14853, USA; jea264@cornell.edu

**Keywords:** ELISA, antibody, non-specific binding, cross-reaction, validation

## Abstract

Different types of enzyme-linked immunosorbent assays (ELISA) have been widely used to control food safety and quality. To develop an accurate and reproducible ELISA, false immunodetection results caused by non-specific binding (NSB) and cross-reaction must be prevented. During the case study of sandwich ELISA development for the detection of porcine hemoglobin (P_Hb_), several critical factors leading to NSB and cross-reaction were found. First, to reduce the NSB of the target analyte, the selection of microplate and blocker was discussed. Second, cross-reactions between enzyme-labeled secondary antibodies and sample proteins were demonstrated. In addition, the function of (3-aminopropyl)triethoxysilane (APTES) was evaluated. Overall, this study highlights the essence of both antibody and assay validation to minimize any false-positive/negative immunodetection results.

## 1. Introduction

Food fraud includes a wide range of deliberate fraudulent acts to foods such as substitution, addition, tampering, dilution, counterfeiting, or misrepresentation of foods or food ingredients, which may cause potential health risks [1,2,3]. Globally, it is estimated that food fraud affects approximately 10% of food products and leads to a loss of approximately USD 10–15 billion each year [4]. Recently, many studies have reported the potential increase of food fraud due to the COVID-19 pandemic [5,6,7]. Among different methods for the surveillance of food fraud, enzyme-linked immunosorbent assay (ELISA) is widely applied due to its advantages of sensitivity, rapidity, selectivity, reproducibility, economy, efficiency, and easiness to handle without complex instruments [8]. In 2019, ELISA accounted for 61% of the total global food safety testing market, and it is a dominant technique for the detection of food adulterants [9]. In addition, ELISA has been widely used in hospitals, clinical laboratories, pharmaceutical companies, and research organizations. The global ELISA market was valued at about USD 1.6 billion in 2018 and is projected to increase significantly at a compound annual growth rate of 5.5% from 2019 to 2028 [10]. The U.S. comprises one of the world’s largest markets [11].

**Figure 1:**
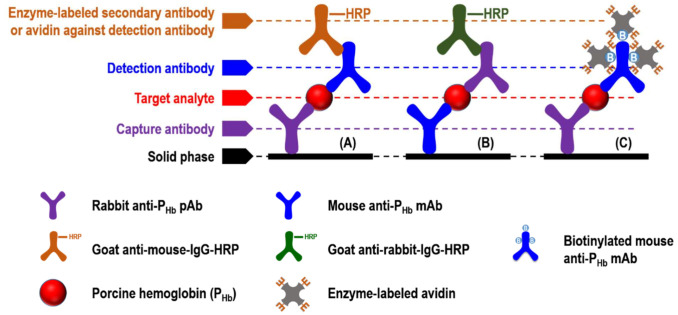
Schematics of indirect sELISA. (**A**) Rabbit anti-P_Hb_ pAb and mouse anti-P_Hb_ mAb was applied as the capture and detection antibody, respectively; (**B**) mouse anti-P_Hb_ mAb and rabbit anti-P_Hb_ pAb was applied as the capture and detection antibody, respectively; (**C**) rabbit anti-P_Hb_ pAb and biotinylated mouse anti-P_Hb_ mAb was applied as the capture and detection antibody, respectively.

In general, sandwich ELISA (sELISA) is one of the formats that can be commercialized due to its standardized quality control and simple operation. Monoclonal (mAb) or polyclonal antibody (pAb) can be used for the capture or detection antibody in sELISA, which can be performed either directly or indirectly. In the direct format, the enzymes- [12], fluorophores- [13], or nanoparticles- [14] conjugated detection antibody enables immunosignal recognition. However, this labeling process could be time-consuming and expensive [13]. In the indirect format (Figure 1), the unlabeled detection antibody can be identified by the labeled secondary antibody (Figure 1A,B). It should be noted that the use of secondary antibodies may lead to cross-reaction, which is defined as any unexpected interaction between a particular antibody and those non-specific antigens [15]. In indirect sELISA, detection antibodies can also be labeled with biotin, which can further interact with enzyme-labeled avidins, such as streptavidin-horseradish peroxidase (HRP) conjugate (Figure 1C).

Accuracy and reproducibility are two of the criteria during assay validation. Accuracy is the degree of closeness of the determined value to the nominal or known true value under prescribed conditions [16]. Reproducibility can be regarded as precision, which is a measurement of the variation in samples in the same assay (within the same run) or different assays (from day to day or from different experimenters) [17]. There are two major factors that affect the accuracy and reproducibility of ELISA. First, during each assay step, any substances may adsorb to the solid phase due to non-specific binding (NSB), causing a high background reading or false immunosignal [17]. For example, NSB of antibodies in sera has been reported by several ELISA studies [18,19,20]. To prevent NSB, blocking is an essential step to saturate the unoccupied sites on the solid phase. To date, few studies have been conducted on the blocking effect using different microplate types. Second, cross-reaction from enzyme-labeled secondary antibody or avidin against detection antibody can reduce the assay selectivity, causing inaccurate and irreproducible findings. For example, cross-reaction between different antibodies and bovine serum albumin (BSA), a commonly used blocker, has been reported [21,22,23]. Therefore, during assay development to quantify porcine hemoglobin (P_Hb_) in raw pork and pork-free meat products to further ensure meat safety and quality [24], we elaborated the importance of studying NSB and cross-reaction in ELISA.

## 2. Materials and Methods

### 2.1. Materials

Two types of 96-well clear polystyrene microplates suitable for immunoassay development, i.e., high-binding (product number: 3590) and medium-binding (product number: 9017), were purchased from Corning Inc. (New York, NY, USA) [25]. BSA suitable for blocking in ELISA applications (A4503), casein sodium salt from bovine milk (CN, C8654), 3,3′,5,5′-tetramethylbenzidine (TMB, 860336), polyethylene glycol (PEG, P2139), anti-mouse immunoglobulin (IgG, Fc specific)-peroxidase antibody produced in goat (goat anti-mouse-IgG-HRP, A2554, RRID: AB_258008, lot No. 069K4789), anti-rabbit IgG (whole molecule)-peroxidase antibody produced in goat (goat anti-rabbit-IgG-HRP, A0545, RRID: AB_257896, lot No. 102M4823), and streptavidin-HRP conjugate from *Streptomyces avidinii* (S5512) were purchased from Sigma-Aldrich (St. Louis, MO, USA). Tween-20 (1706531) was purchased from Bio-Rad Laboratories (Hercules, CA, USA), and (3-Aminopropyl)triethoxysilane (APTES, 123580) was purchased from Beantown Chemical Co. (Hudson, NH, USA). Polyvinylpyrrolidone (PVP, A14315) was purchased from Alfa Aesar (Tewksbury, MA, USA). Non-fat dry milk (NFDM, 0290288705) was purchased from MP Biomedicals, LLC (Solon, OH, USA). Fish gelatin (10976) was provided by Custom Collagen Inc. (Addison, IL, USA). An enzyme inhibitor, Halt Protease Inhibitor Cocktail (78425), was purchased from Thermo Scientific (Rockford, IL, USA). Mouse anti-P_Hb_ mAb was developed at the Florida State University Hybridoma Facility (Tallahassee, FL, USA) [26], and rabbit anti-P_Hb_ pAb was developed at the Hebei Animal Disease Prevention and Control Center (Shijiazhuang, Hebei, China) [24]. Mouse anti-P_Hb_ mAb was biotinylated using EZ-link sulfo-NHS-LC-Biotin (sulfosuccinimidyl-6-[biotin-amido]hexanoate, 21335, Thermo Scientific) according to the manufacturer’s instructions.

### 2.2. Sample Preparation

Three sample models were prepared (Table 1). For *Sample Model 1*, lean meats, including beef steak, chicken thigh, pork loin, pork shoulder, and turkey breast, were purchased from a local grocery store (Tallahassee, FL, USA). Each meat was ground twice using a meat grinder upon receipt. Unless otherwise specified, all extraction was conducted at 4 °C, and centrifugation was performed at 20,000× *g* for 15 min. Briefly, each ground meat was added with three parts (g/mL) of ice-cold extraction solution (12.5 mM NaHCO_3_ and 25 mM NaCl, pH 8.3) containing enzyme inhibitors. For pork loin and pork shoulder, four other extraction ratios (i.e., 1:2, 1:4, 1:5, and 1:10 g/mL) were also performed. After homogenization (11,000 rpm for 2 min, ULTRA-TURRAX T-25 basic homogenizer, IKA Works, Inc., Wilmington, NC, USA), sonication (15 min, Branson Ultrasonic Cleaner, Branson Ultrasonics Corp., Danbury, CT, USA), and end-over-end rotation (1 h), each sample protein extract was centrifuged and filtered.

For *Sample Model 2*, whole bloods from goat, horse, rabbit, and sheep were purchased from LAMPIRE Biological Laboratories, Inc. (Pipersville, PA, USA). Bovine blood was purchased from HemoStat Laboratories (Dixon, CA, USA). Chicken and porcine bloods were collected from local farms (Tallahassee, FL, USA). All whole bloods were 1:100 (mL/mL) diluted using the ice-cold extraction solution and sonicated for 15 min. The supernatant was collected after centrifugation. The Pierce BCA (bicinchoninic acid) Protein Assay Kit (Pierce Biotechnology, Rockford, IL, USA) was used to determine protein concentration, in which BSA was the protein standard (0.025 to 2 mg/mL). For the target analyte (*Sample Model 3*), P_Hb_ (H4131, Sigma-Aldrich) was dissolved in the extraction solution (3 mg/mL), aliquoted, and stored at −80 °C until use.

### 2.3. Indirect sELISA

During assay development, (1) the coating buffer was 10 mM phosphate-buffered saline (PBS, pH 7.2) containing 0–7.5% (mL/mL) APTES; (2) the antibody buffer was 1% (g/mL) of the equivalent blocker (Table 2) containing 0.05% (mL/mL) Tween-20; (3) the added reagent volume was 75 µL/well, while the added volume of each blocker was 200 µL/well; and (4) after each incubation (at least 1 h at 37 °C), at least three washes using the washing buffer (PBST: PBS containing 0.05% (mL/mL) Tween-20) were performed.

**Figure 2:**
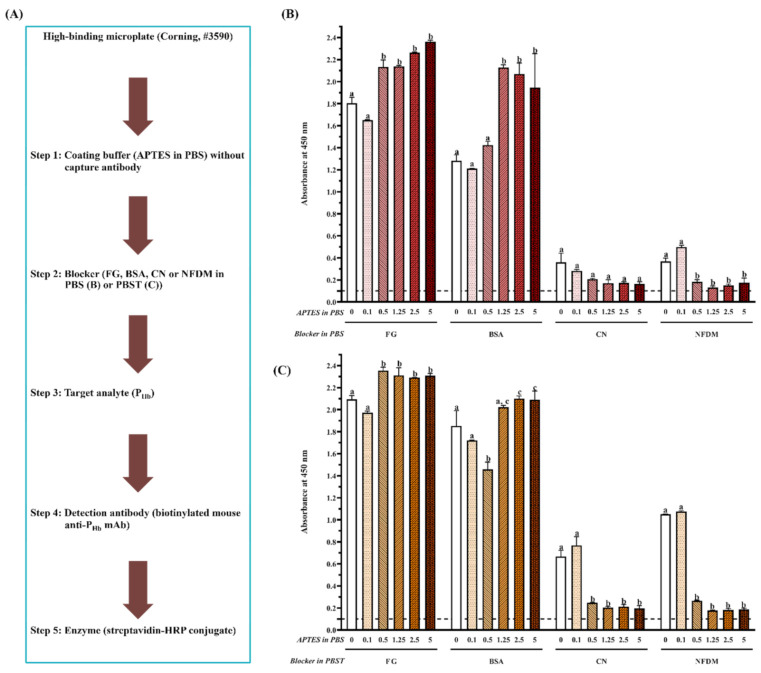
Effect of APTES (0–5%, mL/mL) and blockers on assay NSB using a high-binding microplate. (**A**) Flowchart of experimental protocol; (**B**) blockers were dissolved in PBS; (**C**) blockers were dissolved in PBST. The threshold of the positive absorbance at 450 nm (i.e., A_450_ = 0.1) is shown in the dashed line. FG: fish gelatin; BSA: bovine serum albumin; CN: casein; NFDM: non-fat dry milk. The data are represented as average ± SEM (standard error of the mean, *n* = 2). Different letters within the same blocker indicate a significant difference in absorbance (*p* < 0.05).

**Figure 3:**
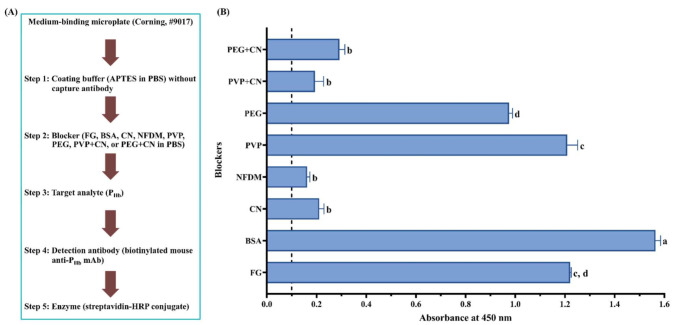
Effect of blockers on assay NSB using a medium-binding microplate. (**A**) Flowchart of experimental protocol; (**B**) blockers were dissolved in PBS. The threshold of the positive absorbance at 450 nm (i.e., A_450_ = 0.1) is shown in the dashed line. FG: fish gelatin; BSA: bovine serum albumin; CN: casein; NFDM: non-fat dry milk; PVP: polyvinylpyrrolidone; PEG: polyethylene glycol. The data are represented as average ± SEM (*n* = 2). Different letters within the group indicate a significant difference in absorbance (*p* < 0.05).

**Figure 4:**
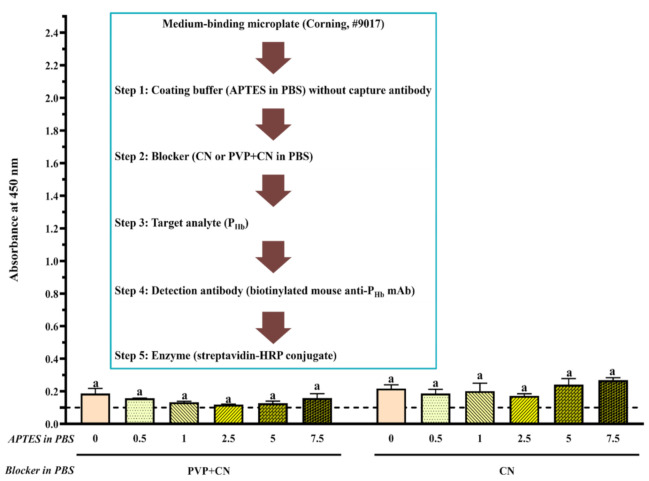
Effect of APTES (0–7.5%, mL/mL) on assay NSB using a medium-binding microplate. The threshold of the positive absorbance at 450 nm (i.e., A_450_ = 0.1) is shown in the dashed line. CN: casein; PVP: polyvinylpyrrolidone. The data are represented as average ± SEM (*n* = 2). Different letters within the group indicate a significant difference in absorbance (*p* < 0.05).

**Figure 5:**
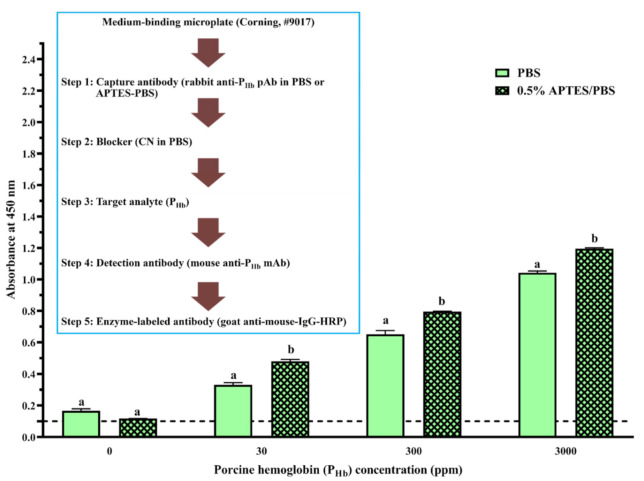
Effect of APTES on the assay immunosignal. The threshold of the positive absorbance at 450 nm (i.e., A_450_ = 0.1) is shown in the dashed line. The data are represented as average ± SEM (*n* = 2). Different letters within the group indicate a significant difference in absorbance (*p* < 0.05).

**Figure 6:**
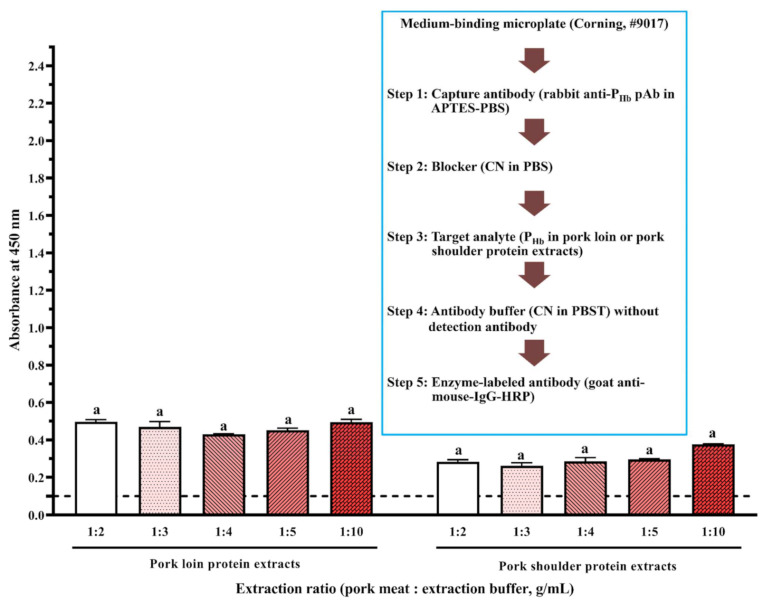
Effect of cross-reaction on the assay immunosignal. The threshold of the positive absorbance at 450 nm (i.e., A_450_ = 0.1) is shown in the dashed line. The data are represented as average ± SEM (*n* = 2). Different letters within the group indicate a significant difference in absorbance (*p* < 0.05).

Briefly, coating buffer without or with capture antibody (3 ppm of mouse anti-P_Hb_ mAb or 1:1000 (mL/mL) diluted rabbit anti-P_Hb_ pAb) was added to the high-binding or medium-binding microplate. After blocking using the selected blockers (Table 2), either P_Hb_ dissolved in the extraction solution (0–3000 ppm) or porcine meat extracts were added. To study the effect of blockers (Figure 2A and Figure 3A) and APTES (Figure 2A and Figure 4) on NSB, each well was added with the detection antibody (1 ppm of biotinylated mouse anti-P_Hb_ mAb) followed by the streptavidin-HRP conjugate (1:1000 (µL/µL) diluted in PBST). To study the effect of APTES on sELISA immunosignal (Figure 5), unlabeled detection antibody (3 ppm of mouse anti-P_Hb_ mAb) followed by goat anti-mouse-IgG-HRP (2.8 ppm) were added. To study the cross-reaction among the capture antibody (mouse anti-P_Hb_ mAb or rabbit anti-P_Hb_ pAb), porcine meat extracts, or enzyme-labeled secondary antibody (Table 3 and Figure 6), either goat anti-rabbit-IgG-HRP (3.4 ppm) or goat anti-mouse-IgG-HRP (2.8 ppm) was added. Color development was performed by adding 0.1 mg/mL of TMB substrate (100 µL/well), followed by incubating at 37 °C in the dark for at least 5 min. The color was stopped by adding 2 M sulfuric acid (25 µL/well), and the absorbance was measured at 450 nm (A_450_) using a microplate reader (BioTek Instruments, Inc., Winooski, VT, USA).

### 2.4. Western Blot

Blood, meat protein extracts, and positive controls (P_Hb_, mouse anti-P_Hb_ mAb IgG, and rabbit anti-P_Hb_ pAb IgG) were separated using a non-reducing sodium dodecyl sulfate-polyacrylamide gel electrophoresis (SDS-PAGE, 4% stacking gel and 15% separating gel) according to Jiang et al. [27] with modifications. Briefly, electrophoretically separated protein bands on the gel were transferred onto a 0.45-µm nitrocellulose membrane (Thermo Scientific). The transferred proteins were visualized using Ponceau S staining. After blocking with BSA-PBS (PBS containing 1% (g/mL) BSA), the membrane was incubated with either biotinylated mouse anti-P_Hb_ mAb (1 ppm) or rabbit anti-P_Hb_ pAb (1:1000 diluted), followed by streptavidin-HRP conjugate (1:1000 (mL/mL) diluted in PBST) or goat anti-rabbit-IgG-HRP (412 ppb), respectively. To study the cross-reaction between enzyme-labeled secondary antibodies and animal proteins, the blotted membrane was directly incubated with either goat anti-rabbit-IgG-HRP (412 ppb) or goat anti-mouse-IgG-HRP (340 ppb).

All antibodies were diluted in BSA-PBST (PBST containing 1% (g/mL) BSA). The incubation time for each step was at least 1 h at room temperature. Between each step, the membrane was washed with PBST several times. The antigens were detected using the luminol chemiluminescence method. The images were captured by Azure c600 Imaging System and analyzed using the AzureSpot software (version 2.0.062).

### 2.5. Statistical Analysis

All experiments were at least duplicated. GraphPad Prism (version 9.0.2 for Windows, GraphPad Software, Inc., La Jolla, CA, USA) was used for data analysis. One-way or two-way ANOVA with Tukey’s test was performed to study (1) the effect of blockers and APTES on NSB of P_Hb_, and (2) the effect of the extraction ratio on the cross-reactive immunosignal. Two-way ANOVA with Sidak’s test was performed to study the effect of APTES on assay immunosignal. *p* < 0.05 was considered as a significant difference.

## 3. Results and Discussion

### 3.1. Antibody Characterization

**Figure 7:**
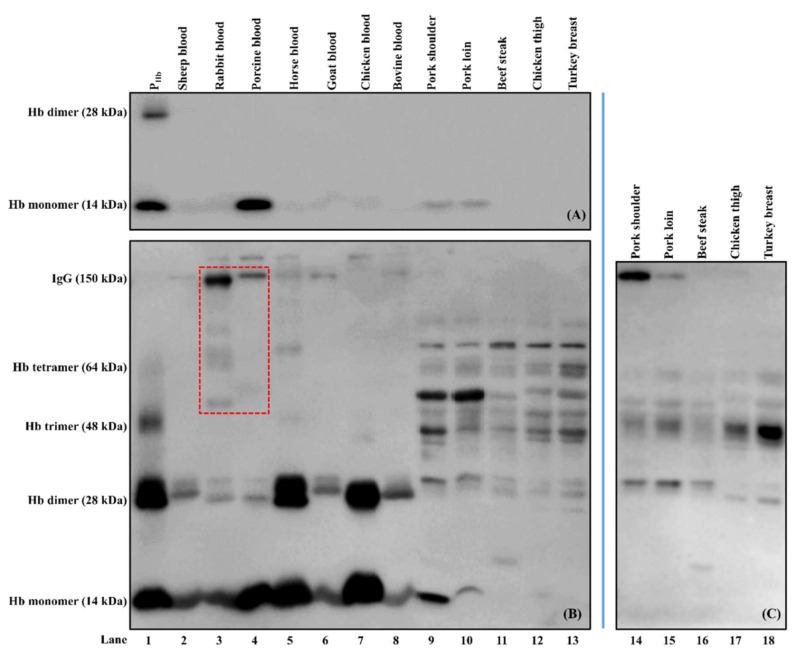
Selectivity of biotinylated mouse anti-P_Hb_ mAb (**A**) and rabbit anti-P_Hb_ pAb (**B**) and cross-reaction between goat anti-mouse-IgG-HRP and meat proteins (**C**). The protein loading mass of each sample was 5 µg/lane except for P_Hb_ (1 µg/lane). IgGs in rabbit and porcine bloods reacting with enzyme-labeled secondary antibody (goat anti-rabbit-IgG-HRP) are indicated using a red dashed box (refer to Figure 8A).

Biotinylated mouse anti-P_Hb_ mAb was specific to P_Hb_ from both porcine blood and meat samples (lanes 1, 4, 9–10, Figure 7A) and did not cross-react with Hb from other species and non-Hb proteins (lanes 2–3 and 5–13). Our results indicate that its selectivity was retained before and after biotinylation [24].

**Figure 8:**
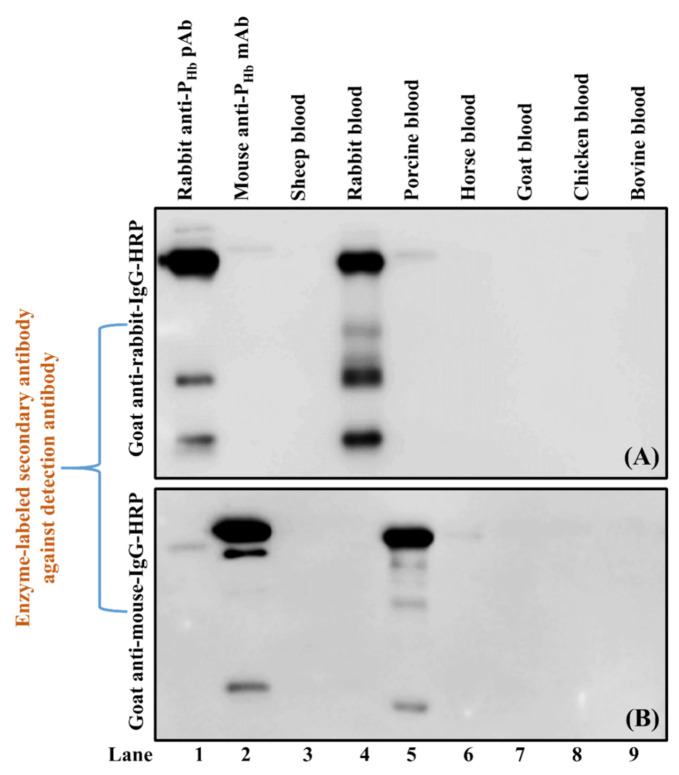
Cross-reaction between IgGs and goat anti-rabbit-IgG-HRP (**A**) or goat anti-mouse-IgG-HRP (**B**). The protein loading mass of each sample was 5 µg/lane except for mouse anti-P_Hb_ mAb (1 µg/lane) and rabbit anti-P_Hb_ pAb (1 µg/lane). Positive controls: rabbit anti-P_Hb_ pAb (lane 1, **A**) and mouse anti-P_Hb_ mAb (lane 2, (**B**)).

As for rabbit anti-P_Hb_ pAb, besides P_Hb_ (lanes 1, 4, and 9–10, Figure 7B), this pAb was non-specific to Hb from sheep, rabbit, horse, goat, chicken, and bovine (lanes 2–3, 5–8). In addition, rabbit anti-P_Hb_ pAb could cross-react with non-Hb proteins (14 kDa to 150 kDa) from both blood and meat samples (lanes 2–13, Figure 7B). It should be noted that goat anti-rabbit-IgG-HRP could also interact with some proteins, such as IgGs in rabbit and porcine bloods (lanes 3–4, red dashed box, Figure 7B), which was confirmed using Western blot (lanes 4–5, Figure 8A).

### 3.2. Non-Specific Binding (NSB)

During ELISA development, in the absence of the capture antibody (Figure 2A and Figure 3A), mainly due to NSB of P_Hb_, the false-positive immunosignals (A_450_ > 0.1) were observed in both high-binding (Figure 2B,C) and medium-binding microplate (Figure 3B). There was no NSB of the detection antibody or enzyme-labeled secondary antibody in a medium-binding microplate (A_450_ < 0.1, Experiments 1–3, Table 3). The effect of the blocker (Tween-20, protein and non-protein component), microplate, and APTES concentration was quantified by the $A_{450}^{\mathrm{NSB}}$ of target analyte (P_Hb_) on the blocked microplate lacking the capture antibody. Overall, the smaller $A_{450}^{\mathrm{NSB}}$, the better blocking effect. The optimal $A_{450}^{\mathrm{NSB}}$ should be closed to that of the chromogen blank since it is the absorbance given by the substrate and stop solution only.

#### 3.2.1. Effect of Tween-20

Tween-20, as a blocker component, significantly increased $A_{450}^{\mathrm{NSB}}$ for each blocker when the APTES concentration was less than 0.5% on a high-binding microplate (*p* < 0.05, Figure 2B,C). Currently, there is controversy from different studies regarding the blocking effect of Tween-20. On the one hand, it was reported to be inefficient in blocking due to its ability of protein desorption [28]. The addition of proteins such as albumin and milk with Tween-20 could increase background noise and reduce ELISA sensitivity [29]. The presence of Tween-20 increased NSB of recombinant phage in ELISA [30], did not block well on the poly-L-lysine treated microplate [29], and even detached the antigens from the microplate [31]. In addition, the interference of Tween-20 with immunoblotting was reported [32]. Other detergents, such as sodium dodecyl sulfate (SDS), Triton, and 3-[(3-cholamidopropyl) dimethylammonio]-1-propanesulfonate (CHAPS), were also not recommended as a blocker component because they not only break target-antibody interaction but also inhibit enzyme-substrate interaction [33]. On the other hand, the blocking effect of Tween-20 has been recognized by some studies. For example, Mohammad and Esen [34] proposed that Tween-20 exhibits the same blocking effect as BSA and NFDM. Low concentrations (0.05–0.1%, mL/mL) of Tween 20 mixed with soymilk proteins could reduce the background absorbance significantly [35]. One possible explanation is that, as a non-ionic detergent, Tween-20 prevents the non-specific hydrophobic interactions while allowing specific antibody interaction with the antigen [34].

#### 3.2.2. Effect of Protein and Non-Protein Blockers

For the high-binding microplate (Figure 2B,C), a similar immunosignal pattern was observed when the same blocker was dissolved in PBS (Figure 2B) or PBST (Figure 2C). Overall, for the same coating buffer, (1) NSB absorbance from FG and BSA was significantly higher than that from CN and NFDM (*p* < 0.05), and (2) there was no significant difference in the blocking effect between CN and NFDM (*p* > 0.05) when the APTES concentration is more than 0.1%. As to the medium-binding microplate (Figure 3B), protein blockers showed a similar immunosignal pattern compared with their counterparts in the high-binding microplate (Figure 2B).

To prevent the false-positive immunosignal caused by NSB of P_Hb_, blocking, as an essential step, can saturate the unoccupied sites with the reagent that does not participate in the immunochemical reactions of the assay [36]. Among four protein blockers, fish gelatin had the worst blocking effect for both microplates, which produced the highest $A_{450}^{\mathrm{NSB}}$. This is because gelatin is more effective in blocking protein-protein rather than protein-plastic interaction [37]. It masks specific sites on proteins and interferes with immunoreactivity, which further results in a higher background and decreased immunosignal [38]. In addition, the lot-to-lot variances could lead to the inaccurate recovery of target analytes [39]. It should be noted that, similar to Tween-20, studies have reported the advantages of gelatin for providing the best positive to negative ratio [40] and improving the ELISA sensitivity significantly [41]. Also, Rajasekariah et al. [42] showed that the blocking efficiency from gelatin increased as a function of its concentration.

BSA was also not efficient, as even a 5% BSA could not inhibit NSB of P_Hb_ on both microplates, which was due to the displacement or loss of BSA in the subsequent steps. Farajollahi et al. [43] reported a displacement of 14% BSA from the well surface after human serum incubation. Ahirwar et al. [44] found the weak binding of BSA to the microplate and its easiness to be washed away using PBST. Studies have demonstrated the possibility of cross-reaction between ELISA reactants and BSA [21,22], which further suggests that a commonly used BSA blocker does not guarantee a good assay performance.

On the contrary, CN provided a better blocking effect than BSA. CN had a very high affinity to plastics and a low affinity to other proteins and was considered the most effective blocker [37]. In addition, CN variants have a molecular weight from 19 kDa to 25 kDa [45], which is able to prevent blocking leakiness caused by the penetration of other reagents [46]. Grogan et al. [47] noted that CN could reduce NSB by 86% compared to 46% by BSA. The blocking efficiency of CN over other blockers, such as BSA and gelatin, has been reported in many studies [42,48,49]. In addition, CN generally does not require a high concentration, as a 1% CN should be enough to achieve the optimal blocking effect.

NFDM exhibited a similar blocking effect to CN due to its molecular diversity and amphipathic characteristics of milk proteins [49]. A 10% NFDM offered a better blocking effect than 10% BSA and 20% egg albumin [50]. The superiority of NFDM in ELISA blocking was also demonstrated by Akerstedt [51] and Huber et al. [52]. Despite the advantages of low cost, good blocking efficiency, and readily available dispersibility, NFDM tends to deteriorate rapidly if not stored properly. It was not applicable to detect phosphorylated proteins [35] and was reported to contain inhibitors for biotin-streptavidin interaction, in that high concentrations of NFDM could decrease assay sensitivity [53]. Studies have also reported its inability for blocking in the ELISA procedure of S100 protein [54].

When a non-protein blocker (PVP or PEG) was used individually, it did not exhibit a good blocking effect (Figure 3B). Although a combination of CN significantly improved its blocking effect (*p* < 0.05), it was not statistically different from CN used alone (*p* > 0.05). This is because these non-protein blockers are polymers that are known for their ability to coat hydrophobic surfaces [36,55]. The usage of PEG improved both positive and negative signals, which led to a decreased assay sensitivity [40]. Their blocking deficiency was also reported by Huber et al. [52], who found that PVP and PEG could not replace protein blockers in the ELISA development of food allergen detection. It is recommended to combine the polymers with protein blockers to achieve the desired blocking effect [36]. Due to the high viscosity of the polymers, they are commonly used at a low concentration [56].

#### 3.2.3. Effect of Microplate Type

The $A_{450}^{\mathrm{NSB}}$ from the high-binding microplate was 71% and 127% higher than that from medium-binding microplate blocked by CN and NFDM, respectively (Figure 2B and Figure 3B). Microplate selection is a critical step during ELISA development [25,57,58]. Generally, two types of polystyrene microplate, i.e., high-binding and medium-binding, are commonly used. Our study showed that the false-positive immunosignal from NSB of P_Hb_ was much higher in the high-binding microplate than that from the medium-binding microplate. The high-binding surface (negatively charged) is designed to bind medium molecules (10 kDa–20 kDa) through both ionic and hydrophobic interactions. The non-treated or medium-binding surface is hydrophobic in nature and is able to absorb large molecules (>20 kDa), such as an antibody, through passive interaction [59]. The high-binding or medium-binding microplate has a binding capacity of 100–200 ng or 500 ng of mouse IgG/cm^2^, respectively [59]. The higher binding capacity could not only improve assay performance but also potentially increase NSB. Gibbs et al. [36] reported that the high-binding microplate was more challenging to block than the medium-binding surface. The high-binding surface could not be effectively blocked using a non-ionic detergent alone, which required the incorporation of protein blockers [60].

In addition, the NSB was mainly caused by the target analyte (P_Hb_) instead of the detection antibody or enzyme-labeled secondary antibody, which was probably due to the following three reasons. First, P_Hb_ monomer (15 kDa) could penetrate slots more easily because it has a relatively small size compared to antibodies. Second, P_Hb_ is positively charged due to its isoelectric point (pI 7.1) lower than the pH of the extraction solution (pH 8.3), which facilitates its attachment to the high-binding microplate through ionic interactions [61]. Third, a high concentration of P_Hb_ (3000 ppm) also contributes to NSB [43]. It should be noted that, although NSB of antibodies was not encountered in this study, it has been reported by other researchers [20,43].

#### 3.2.4. Effect of APTES during Coating

On the high-binding microplate, using CN or NFDM blockers, compared to 0% APTES, NSB of P_Hb_ from a 0.5% APTES-treated microplate decreased at least 40%, while the increase of APTES concentration did not produce a more desirable blocking effect (Figure 2B,C). On the medium-binding microplate, using CN or PVP + CN blockers, no significant difference in NSB absorbance was observed as a function of APTES concentration (*p* > 0.05, Figure 4). In addition, when the medium-binding microplate was incubated in a solution of 0.5% APTES/IgG in PBS, the immunosignal increased by 45%, 22%, and 15% at 30 ppm, 300 ppm, and 3000 ppm of P_Hb_, respectively (Figure 5).

APTES, as a coupling agent, can adsorb to the negatively charged high-binding microplate via electrostatic interactions in PBS [62]. Multilayers of APTES are further formed through electrostatic interactions and/or hydrogen bonding [62,63]. It is hypothesized that this multilayered structure may contribute to the reduction of NSB on the high-binding microplate. Since the medium-binding microplate is hydrophobic, such reduction was not observed. In addition, in the presence of APTES, water molecules in PBS render the electrostatic interaction between the amine groups from APTES and the carboxyl group in the IgG, which form a stable APTES-antibody polymer network [64]. Due to the slight hydrophobicity of the aliphatic chain from APTES [65], a better immobilization of APTES-antibody polymer was reported on a polystyrene surface [66], leading to a higher immunosignal in ELISA. The enhancement of ELISA sensitivity using an APTES-treated microplate has been previously reported [63,66,67]. Overall, APTES could decrease NSB depending on the type of microplate and blocker and improve the assay immunosignal.

### 3.3. Cross-Reaction

#### 3.3.1. Cross-Reaction of the Enzyme-Labeled Secondary Antibody and Unintended IgGs

Two cross-reactions, i.e., between rabbit anti-P_Hb_ pAb IgG and goat anti-mouse-IgG-HRP (Experiment 4, Table 3) and between mouse anti-P_Hb_ mAb IgG and goat anti-rabbit-IgG-HRP (Experiment 5, Table 3), were verified using Western blot by including rabbit anti-P_Hb_ pAb IgG (lane 1, Figure 8A) and mouse anti-P_Hb_ mAb IgG (lane 2, Figure 8B) as positive controls. Our results confirmed that goat anti-rabbit-IgG-HRP could weakly cross-react with mouse and porcine IgGs (lanes 2 and 5, Figure 8A), while goat anti-mouse-IgG-HRP could falsely immunodetect IgGs from rabbit, porcine, and horse (lanes 1, 5 and 6, Figure 8B).

Mainly due to the IgG resemblance from different animal species [68], cross-reaction between an enzyme-labeled secondary antibody and the antibody(ies) used in the previous step has been reported in different studies [69,70]. To avoid this misusage, some commercial secondary antibodies indicate their potential species cross-reactivity. For example, the cross-reactivity information of Goat Anti-Mouse IgG Biotinylated Antibody (BAF007, RRID: AB_355776, R&D Systems, Inc., Minneapolis, MN, USA) is listed in the product details [71], which is helpful during research design. To reduce the cross-reactivity from the secondary antibody, an additional purification process, i.e., cross-adsorption (also referred to as pre-adsorption), can be adopted [72]. This approach reduces or even eliminates IgG cross-reactivity with other undesired species, improves antibody selectivity, and has been used to produce different commercial labeled-secondary antibodies [73,74,75].

#### 3.3.2. Cross-Reaction among Capture Antibody, Non-P_Hb_ Proteins, and Enzyme-Labeled Secondary Antibody

In the absence of detection antibody, a false-positive immunosignal caused by the cross-reaction among rabbit anti-P_Hb_ pAb, porcine meat proteins, and goat anti-mouse-IgG-HRP was identified (Figure 6). Since there was no NSB of porcine proteins to the medium-binding microplate ($A_{450}^{\mathrm{NSB}}$ < 0.1), this false-positive immunosignal was mainly due to non-P_Hb_ proteins in porcine meats non-specifically immunodetected by rabbit anti-P_Hb_ pAb and goat anti-mouse-IgG-HRP at the same time. For the same meat origin, an increase in the protein extraction ratio could not reduce the negative impact of cross-reaction (Figure 6). At the same extraction ratio, the immunosignal from pork loin was significantly higher than that from pork shoulder (*p* < 0.05), suggesting that the amount of cross-reactive proteins is location-dependent [24]. Since this assay had both a capture antibody and an enzyme-labeled secondary antibody, it can be considered as a direct sELISA. Western blot was used to identify the cross-reactive proteins that could be simultaneously detected by these two antibodies. It is hypothesized that one or more non-P_Hb_ proteins (37 kDa to 70 kDa) were immunodetected by both rabbit anti-P_Hb_ pAb (lanes 9–10, Figure 7B) and goat anti-mouse-IgG-HRP (lanes 14–15, Figure 7C), which led to this false-positive immunosignal in ELISA. It should be noted that this cross-reaction was also observed in non-porcine species, including beef, chicken, and turkey (lanes 11–13, Figure 7B and lanes 16–18, Figure 7C).

The cross-reaction in ELISA leads to the under- or overestimation of target analyte concentration. In this study, the endogenous non-P_Hb_ proteins in the tested sample cross-reacted with both capture and enzyme-labeled secondary antibody. Cross-reaction occurred because these proteins have similar epitopes to P_Hb_ and IgGs, which are able to bind to both antibodies. It is commonly accepted that pAbs are more cross-reactive than mAbs since they are secreted from different plasma cells and are capable of recognizing multiple epitopes [76]. For example, Yu et al. [77] identified the cross-reactivity of laboratory-produced rabbit anti-shrimp tropomyosin pAb with tropomyosin from crab and clam. Evaluating antibody cross-reactivity is a critical validation experiment. Although there are no universal criteria for antibody validation, many valuable suggestions are available [78,79,80]. It is recommended that the antibody properties such as selectivity and reproducibility should be periodically studied in context [80]. This is because the changes of assay format or parameters may alter antibody/assay characteristics over their shelf life. During the manuscript preparation, it is encouraged to provide antibody information such as the catalog number, lot number, and Research Resource Identifiers (RRIDs) [78,79,80].

In addition, researchers should be aware of the biotin residues in the analyzed sample, which can produce a false signal when streptavidin-HRP conjugate is used in ELISA. Biotin can be found in various foods. For example, approximately 416 ng/g of biotin can be found in animal meat [81]. The U.S. Food and Drug Administration (USFDA) has addressed the concern of biotin interference in causing false-negative results [82]. The negative impacts from exogenous biotin on immunoassay performance have been reported, especially in the clinical immunoassays [83,84]. At the current stage, it is difficult to predict and quantify the biotin interference [85]. Several strategies to reduce biotin interference, such as (1) testing the sample using another platform other than the streptavidin-HRP ELISA, (2) sample pretreatment using streptavidin, and (3) diluting the sample, provided that the analyte concentration will not fall below the assay detection limit, can be adopted during assay development [86].

## 4. Conclusions

Using the development of indirect anti-P_Hb_ sELISA as a case, the importance of studying NSB and cross-reaction in ELISA to prevent false findings was illustrated. In this study, the NSB of P_Hb_ was a particular issue for the high-binding microplate in that none of the tested blockers showed the desired blocking effect. The NSB was reduced on the medium-binding microplate when CN and NFDM were applied. BSA, as a commonly used blocker, did not block the unoccupied sites well. Other ingredients, including gelatin, PEG, PVP, and Tween-20, also showed the blocking deficiency. The incorporation of APTES in the coating buffer not only decreased the NSB immunosignal but also improved the assay sensitivity due to the formation of multilayers through electrostatic interactions and/or hydrogen bonding. In addition, the cross-reaction between non-target proteins and antibodies led to a false-positive immunosignal in sELISA, which was further confirmed by Western blot.

Therefore, to ensure assay accuracy and reproducibility, a specific location on the microplate should be designated to study NSB and cross-reaction. For NSB, wells are coated with corresponding coating buffer followed by routine blocking, adding samples, and detection procedure. An optimal blocking should not induce any NSB absorbance. An ideal blocker should be decided according to the microplate, assay format, and target analyte properties. For cross-reaction, the non-specific interaction between unintended IgGs and secondary antibodies, together with the interaction between the non-target and antibody, should not be underestimated. It is recommended to provide as many antibody information as possible to ensure that any antibodies used in their research can be unambiguously identified during immunoassay development.

## Figures and Tables

**Table 1:** Summary of three sample models.

Sample Preparation Steps	*Sample Model 1*	*Sample Model 2*	*Sample Model 3*
	Meat Protein Extracts	Whole Blood Protein Extracts	P_Hb_ (H4131, Sigma-Aldrich)
1. Extraction/dissolving (12.5 mM NaHCO_3_ and 25 mM NaCl, pH 8.3)	1:2, 1:3, 1:4, 1:5, 1:10 (g/mL) *	1:100 (mL/mL)	3 mg/mL
2. Homogenization (11,000 rpm, 2 min)	Yes	No	No
3. Sonication (130 W, 15 min)	Yes	Yes	No
4. End-over-end rotation (1 h, 4 °C)	Yes	Yes	No
5. Centrifugation (20,000× *g*, 15 min, 4 °C)	Yes	Yes	No
6. Protein concentration determination (BCA assay)	Yes	Yes	No
7. Relevant figures	6 and 7	7 and 8	2, 3, 4 and 5

* Extraction solution containing enzyme inhibitors.

**Table 2:** Blockers used in indirect sELISA.

Blockers	Component I	Concentration (%, g/mL)	Component II	Concentration (%, g/mL)
*Protein-based*				
BSA	Bovine serum albumin	5		
CN	Casein	1		
NFDM	Non-fat dry milk	5		
FG	Fish gelatin	1		
*Non-protein-based*				
PEG + CN	Polyethylene glycol	5	Casein	1
PVP + CN	Polyvinylpyrrolidone	5	Casein	1

**Table 3:** Effect of NSB and cross-reaction on indirect sELISA using a medium-binding microplate.

Experiment No.	1	2	3	4	5
Schematics	Figure 1B	Figure 1B	Figure 1C	Figure 1A	Figure 1B
Objectives	Non-specific binding			Cross-reaction	
Step 1: Capture antibody in PBS	None			Rabbit anti-P_Hb_ pAb	Mouse anti-P_Hb_ mAb
Step 2: Blocker	CN in PBS				
Step 3: Target analyte (P_Hb_)	None				
Step 4: Detection antibody in antibody buffer (CN in PBST)	None	Mouse anti-P_Hb_ mAb	Biotinylated mouse anti-P_Hb_ mAb	None	None
Step 5: Enzyme-labeled antibody against detection antibody in antibody buffer	Goat anti-mouse-IgG-HRP (RRID: AB_258008)		None	Goat anti-mouse-IgG-HRP (RRID: AB_258008)	Goat anti-rabbit-IgG-HRP (RRID: AB_257896)
			Streptavidin-HRP conjugate in PBST		
A_450_ (mean ± SEM)	0.044 ± 0	0.050 ± 0.003	0.050 ± 0.003	0.176 ± 0.007	0.644 ± 0.013

## Data Availability

Not applicable.
